# Health and Economy in COVID-19 Era: A Plan for Reconstituting Long-Term Economic Security

**DOI:** 10.3389/fpubh.2020.00235

**Published:** 2020-05-27

**Authors:** Mary Beth Allen, Mehdi Mirsaeidi

**Affiliations:** ^1^Division of Pulmonary and Critical Care, University of Miami, Miami, FL, United States; ^2^Department of Public Health Sciences, University of Miami, Miami, FL, United States

**Keywords:** health, economy, immunity, COVID-19, pandemic

## Abstract

COVID-19 is a rapidly evolving pandemic, which represents a multifaceted global threat. Given the economic consequences, most researchers agree that social distancing measures are an effective strategy relative to the cost. Previous studies indicate that community size as well as viral population risk groups should be considered in forming an effective targeted social distancing strategy. The resultant delay in the occurrence of infections in order to support vaccine development has been shown to be an effective policy. However, a return to normalcy from the current situation would require policy intervention that transforms the American economy along with continued targeted social distancing and the use of medical science as a tool to facilitate gradual personal interactions of low-risk individuals. We believe that the adoption of rapid IgG testing would be best suitable for widespread population-level screening as part of a comprehensive plan for incrementally rebuilding the in-person workforce. As such, this crisis represents an opportunity for the United States to increase automation of the manufacturing sector, shrink supply chains, and create higher-level jobs in order to reduce the dependency on other countries for critical supplies. This economic transition to better utilize technology along with reconstruction of the workforce could improve the standard of living for many Americans as well as better prepare the US for future pandemics.

## Introduction

A pandemic represents a multifaceted global threat. In addition to the obvious impact on population health, pandemics also represent an important threat to the global economy. These important economic costs can potentially result in a comparable impact on population health, as all economic factors have a proven positive correlation with key health status outcomes (1). Therefore, effective public health policy must always respect the impact of economic factors on population health as health and economic stability remain as variables in a reinforcing feedback loop.

## Health and Economics

The relationship between economic health and physical health is well–described. Social determinants of health combines social and economic status based on a combination of race, level of education, level of income, and community, which defines the characteristics of a person's life that drive health related decisions and responses and determines the ability of an individual to both move up or down the socioeconomic ladder. These implications emphasize the importance of the economy in improving health outcomes through upward social mobility, and the importance of fair access to economic benefits. Economic justice was a key issue of debate with the stimulus package that provided more than $2 trillion dollars in aid for the American economy. With bipartisan support, the bill eventually passed with concessions to American families that include direct stimulus payments and expanded safety net protections including paid time off, expanded unemployment insurance and debt relief.

### Social Distancing Policie

In the absence of proven treatment or vaccination, social (physical) distancing is the most proven and effective means of combating communicable infectious diseases. For COVID-19 impacted regions worldwide, this remains one of the few options to slow the spread of the infection while preserving fragile health delivery institutions. Social distancing policy usually includes school and business closures and home-based work recommendations, while enforced curfews and quarantine orders have also been enacted. Although studies acknowledge the economic consequences of forced closures of business and restrictions on private businesses, most researchers agree that social distancing measures are effective relative to cost as part of pandemic preparedness (2). Further, the burden of disease is well-noted to impact the economy without social distancing policy with many economists arguing that preserving lives has a shorter term economic impact. However, not all studies agree on the appropriate iteration of social distancing as clear policy depends on unique features of the virus as well as geographical features and risk groups for severe disease and mortality. In a simulation study of a community of 10,000 residents, targeted social distancing focusing on children and teenagers was found effective for the influenza pandemic occurring in the 1950s through school closures and home-based education. Simulations similar to the Spanish influenza epidemic identified that more aggressive targeting was suitable involving children through young adult populations. These findings indicate that community size as well as viral population risk groups should be considered in forming effective targeted social distance policy (3). Because resource utilization remains a concern for policy makers in the event of a pandemic, social distancing measures as a method for reducing the demand for a limited stockpile of antivirals was not found effective over a long period of time, but delaying the occurrence of infections in order to support vaccine development was found to be effective policy (4).

### Disease Characteristics and Treatments

The implications of the literature applicable to the current outbreak of the Coronavirus disease 2019 (COVID-19) leave many questions. Because COVID-19 is caused by a novel corona virus, many of the disease features researchers anticipated are vastly different and not well-understood. Among these features are differing presentations of disease and severity within populations. While early data suggest that older populations were at an increased risk for severe disease, the identification of infections in different populations has revealed the presence of severe disease in much younger populations. Also, no proven medication protocol currently exists, which has resulted in clinical trials all over the world being conducted to create new therapies, develop a vaccine, and evaluate existing medications developed for the treatment of other viral illnesses. With the latter representing the most immediate benefit with some data also understood and an existing supply and distribution chain in place, several of these therapies have been tested in both laboratory and clinical settings with mixed results. With remdesivir showing the most promising results so far of existing antivirals, the US Food and Drug Administration has recently issued emergency authorization for the experimental use in treatment of severe COVID-19 (5). This will create a practical challenge as the demand for the drug will undoubtedly exceed the existing supply, and the continued interruption of global supply chains will delay immediate production and distribution to care centers and patients in need. Despite this, the promise of remdesivir is encouraging following the disappointing results from hydroxychloroquine studies, which found unacceptable side-effects with no significant effect on disease outcome (6, 7). Still, shortages of hydroxychloroquine were immediate due to widespread experimental use for both COVID-19 patients as well as prophylaxis for at risk healthcare workers. These shortages had important consequences for patients who receive the medication for the management of chronic autoimmune disorders who were unable to access their regular treatment. Further, prolonged mechanical ventilation as the only validated treatment option in severe COVID19 may likewise present scarcity challenges as has occurred in Italy and Spain. Furthermore, the reinfection rate seems to be low, which has important implications for the evolving immunity of the herd in the future. Existing social distancing policy in many states has minimized the interaction of American communities, states and regions that is likely to support variations in peaks between communities. According to the Institute for Health Metrics and Evaluation (IHME), several states with a high volume of cases are expected to have a prolonged epidemic peak for multiple weeks from early May to June, with the exception of New York, which attained peak infections early-mid April. This projection has been calculated based on the current social distancing measures and the varying degrees of population density, which will result in outbreaks in different communities occurring at different times. This has important implications for the fragile healthcare delivery system as shifting resources to the areas in the most need of equipment, medical staff, and other important resources will only be possible if epidemic peaks do not occur in unison.

## Discussion

The return to normalcy will only occur through a complex assortment of policy interventions that transform the American economy, continue targeted social distancing, and use medical science as a tool to personalize social distancing to facilitate gradual personal interactions of low risk individuals.

Among the many differences in the COVID-19 pandemic with previous influenza pandemics, one of the most notable is the ability to facilitate operations through technology. Many industries operating in professional services and functional departments within companies like management and administration have already adopted conferencing technology that facilitate in person interactions. Similar implementations of technology have not been achieved in manufacturing and distribution as low level employment has driven these functions that are now considered essential. Further, the impact of the global economy most specifically from the burden of disease in China has placed entire supply chains at risk for critical medical supplies. The US should use this crisis as an opportunity in invest in the automation of the manufacturing sector, shrink supply chains, and create higher level jobs as a benefit to the economy. Included in this policy should be workforce training available to those who have been replaced through automation.

Social distancing policies should continue as each region, state, and city locality make decisions regarding the reopening of businesses based on their unique disease curve. This has already happened as a result of unclear federal policy, but the practice of each community should be standardized. Workplaces should be adapted to this policy and facilitate standardization of physical distancing.

Medical science will support the gradual reconstitution of the in person work force. This step will depend on many factors such as the development of treatment protocol, vaccine development, and currently available antibody testing that identifies individuals with COVID-19 immunity who can re-enter these industries without posing the risk of infection or infecting others. For those previously infected with COVID-19 infection, severe acute respiratory syndrome coronavirus 2 (SARS-CoV2) immunity develops with an immunoglobulin response measurable by increasing IgM and IgG levels that have been shown to eliminate viral load and promote recovery (8). Although antibody testing is available now, the adoption of rapid IgG testing would be best suitable for widespread population level screening as part of a comprehensive plan for incrementally rebuilding the in person work force.

The human cost of this pandemic is without question significant. However, the transitioning of the economy to better utilize technology along with reconstructing the work force could improve the standard of living for many Americans as well as better prepare the US for future pandemics. We could also create a better response annually to seasonal influenza as its annual cost to the US in terms of morbidity, mortality and loss in quality adjusted life years is significant. Finally, the political support for prioritizing public health preparedness as a matter of national and economic security will likely be stronger and will result in better surveillance that identifies and prevents future pandemics, and streamlines strong national and coordinated responses that will mitigate the potential damage to the economic and health infrastructure and support a more timely recovery.

## Data Availability Statement

All datasets generated for this study are included in the article/supplementary material.

## Author Contributions

MM and MA were involved in conception, design, and drafting of the manuscript. MM was involved in revision of the manuscript for important intellectual content.

## Conflict of Interest

The authors declare that the research was conducted in the absence of any commercial or financial relationships that could be construed as a potential conflict of interest.
